# Effectiveness and feasibility of a motivational interviewing intake (MII) intervention for increasing client engagement in outpatient addiction treatment: an effectiveness-implementation hybrid design protocol

**DOI:** 10.1186/s13722-023-00412-y

**Published:** 2023-10-21

**Authors:** Margo C. Hurlocker, Theresa B. Moyers, Melissa Hatch, Geoffrey Curran, Barbara McCrady, Kamilla L. Venner, Katie Witkiewitz

**Affiliations:** 1 Department of Psychology, University of New Mexico, Albuquerque, NM 87131 USA; 2 Center on Alcohol, Substance Use, and Addictions, University of New Mexico, Albuquerque, NM 87106 USA; 3 Department of Pharmacy Practice, College of Pharmacy, University of Arkansas for Medical Sciences, Little Rock, AR 72205 USA; 4 Health Services Research and Development Service, Center for Mental Healthcare and Outcomes Research, Central Arkansas Veterans Healthcare System, North Little Rock, AR 72114 USA; 5 Department of Psychiatry, College of Medicine, University of Arkansas for Medical Sciences, Little Rock, AR 72205 USA

**Keywords:** Attrition, Substance use disorder treatment, Behavioral intervention, Implementation feasibility, Effectiveness

## Abstract

**Background:**

Client discontinuation from outpatient addiction treatment programs is common, and the initial intake is the service delivery point with the highest attrition rate. Replacing the comprehensive intake assessment with a person-centered Motivational Interviewing (MI) intervention is a potential solution to address provider and client concerns about the disengaging, time-intensive nature of the typical initial intake. It remains unclear whether the use of an alternative to the standard intake at the initial visit can fit within typical organizational reporting requirements, whether it decreases attrition, and whether implementation of person-centered intake procedures within outpatient addiction treatment programs is feasible, acceptable, and can be sustained.

**Purpose:**

To describe the methods and design of an effectiveness-implementation hybrid type 1 trial of a Motivational Interviewing at Intake (MII) intervention using the Consolidated Framework for Implementation Research (CFIR).

**Methods:**

The study will determine the effectiveness of two intake conditions: (1) standard comprehensive intake assessment (intake-as-usual [IAU]), and (2) MII consisting of a person-centered discussion between provider and client about the client’s desire and intent to enter treatment. Although both interventions are focused on understanding client presenting complaints and needs for treatment, the delivery differs as the IAU uses a semi-structured assessment guide, while MII applies the theory of MI to have a conversation about treatment engagement. Adults seeking outpatient addiction treatment services will be randomly assigned to the MII condition (n = 75) or the IAU condition (n = 75). Primary outcomes will be client engagement (i.e., treatment entry, attendance, and completion) obtained from the electronic medical record. Secondary outcomes (client motivation and therapeutic alliance) will be putative mechanisms of client engagement assessed immediately before and after the intake. The trial also will explore determinants of effective, sustainable implementation using assessments of organizational readiness and capacity to change, as well as interviews on MII implementation feasibility.

**Conclusion:**

This trial of an MII intervention will investigate the feasibility of a motivational intervention as an initial contact with substance use treatment-seeking clients as well as indicators of intervention effectiveness within the systems where it is employed.

*Trial registration* Clinicaltrials.gov identifier: NCT05489068

## Contributions to the literature

- Research demonstrates that the initial intake is the service delivery point when most clients discontinue addiction treatment. For many clients and providers, a client-centered intervention can be more engaging and applicable.
- Motivational interviewing (MI) is an evidence-based intervention that promotes client engagement and commitment to make choices consistent with personal values and goals, and the client-centered format may also capture intake information required of external agencies.
- This study will use an effectiveness-implementation hybrid design to examine whether an MI at intake (MII) intervention increases client engagement and to identify the determinants of implementing MII within addiction treatment programs.

## Background

Substance use disorders (SUDs) are prevalent and pervasive chronic problems that can result in myriad personal and societal consequences. In 2020, 40.3 million individuals aged 12 and older (14.5% of the United States population) had an SUD [1], almost twice the number of individuals in 2019 (20.4 million or 7.4%) [2]. Health and social costs associated with SUD exceed $600 billion each year [3]. Although addiction treatment has demonstrable benefits in reducing symptoms and offsetting the costs of SUD, individuals rarely choose to attend formal treatment and then commonly discontinue attending treatment prior to treatment completion, which heightens client risk of returning to harmful substance use and exacerbates treatment facility costs. There are multiple points when a client may disengage from treatment, but 44% of clients do not return after the initial intake [4], making this the service delivery point with the highest attrition rate. Although circumstances beyond the treatment facility likely influence client disengagement [5], understanding which aspects of the initial intake may contribute to client disengagement and developing ways to improve the intake process may help promote client engagement and retention in addiction treatment.

The initial intake session is quite similar across addiction treatment programs, with most outpatient programs (i.e., 93%) [6] tasking a clinical provider with conducting a comprehensive intake assessment to inform treatment planning (“Information-First”). With guidance from the American Society on Addiction Medicine (ASAM), the comprehensive intake assessment asks about the diverse problems individuals experience in relation to their substance use and helps to match clients to their appropriate level of care [7]. Further, the comprehensive intake assessment is an efficient way to gather client information required by health insurance and other funding agencies to determine client eligibility for services. Despite these contributions, clients and providers alike have reported concerns regarding the length, focus, and purpose of the intake assessment, which may contribute to clients not returning to treatment. Specifically, the focus on fact-gathering during intake undermines exploration of the reasons that lead clients to seek treatment in the first place [8]. Relatedly, clients and providers identify the lack of rapport as an impetus for early disengagement [9, 10], precisely because the intake assessment does not address the client’s most relevant concerns. Clients who initiate addiction treatment prefer being included in treatment decisions, working with staff who are empathic, and feeling autonomous in their choices [11–13]. Providers and administrators also are frustrated with an Information-First intake structure, noting that this type of first contact is not engaging, is unacceptably long, and is comprised of excessive, redundant paperwork and questions that are intended largely to meet agency, not client, needs [14–16]. In short, a comprehensive intake assessment does not achieve the primary goal of the initial intake: client engagement.

Given that clients tend to seek treatment when their lives have become unmanageable, it is critical to explore why the client wants treatment *now* during their initial contact with a treatment agency. Despite empirical support for a patient-oriented, recovery-based model of care [17–19], addiction treatment programs have made few, if any, changes to their intake services. The primary source of tension may be that most programs are beholden to state/federal agencies and health insurance companies, all of which require a wealth of client information at the outset to provide financial support or reimbursement [20]. Unfortunately, most agencies are not able to offer a broad variety of treatments suitable to the problems that are carefully identified during this intensive intake process. Further, referral sources also are usually limited, resulting in a detailed assessment of problems that cannot be readily addressed.

Previous research focused on adapting intake procedures to focus more on client engagement in addiction treatment programs has tested the value of adding Motivational Interviewing (MI) to the intake assessment. These interventions are often referred to as “incorporating” or “integrating” MI. The result has often been a null effect on client engagement and retention [21–26]. One reason may be how MI was implemented into the intake process. Specifically, some researchers provided a comprehensive assessment *prior* to delivering MI whereas others delivered a comprehensive assessment *within* an MI framework; both methods led to inconsistent client engagement outcomes. No prior work has tested the impact on client engagement rates as a *replacement* to the comprehensive assessment. Another reason for the inconsistency may be the variable format of the MI interventions. Studies either integrated components from other evidence-based interventions into the MI intervention (e.g., personalized normative feedback [27]) or they included fact-gathering questions in the MI intervention [28]. Adding non-MI components to an MI intervention precludes identifying what aspects of the intervention led to the mixed findings so commonly seen in the MI literature [29]. This is an important limitation that is likely due to the rapid, widespread dissemination of MI [30], with minimal attention to its effective, sustainable implementation into standard practice.

It may be important to change the entire intake process, rather than build in MI to the currently implemented lengthy intake process. Changing the intake process for addiction treatment may also require broader changes in programs, further complicating the adoption potential of a new intake approach. For example, the workflow may change such that more paperwork may be required either before or after intake rather than during the intake when the provider is attending carefully to the client. Modifying the Information-First approach also requires re-training personnel, which may be difficult to initiate and sustain. Prior to adapting intake procedures, it is critical to first determine the effectiveness and feasibility of replacing existing intake procedures with an alternative intake process. With an evidence base, findings could be used to encourage external agencies to modify their requirements of an agency’s first contact with clients.

The current trial will utilize an effectiveness-implementation hybrid type 1 (HEI-1) design to evaluate the clinical effectiveness of a Motivational Interviewing at Intake (MII) intervention to increase client engagement, retention, and success in treatment, as well as two theoretical mechanisms of engagement (client motivation and therapeutic alliance). Simultaneously, the trial will examine perceptions among personnel of facilitators and barriers to the effective and sustained implementation of the MII intervention. Given that clients and providers alike recommend improving an intake appointment in a manner that fosters therapeutic rapport and encourages client engagement, MI may be optimal, with demonstrated evidence to increase client engagement and commitment to make behavioral changes [31]. In contrast to prior efforts to integrate MI into standard intake assessment, this trial will test whether *entirely replacing the standard intake assessment with an MII intervention* can promote client engagement. An evaluation of the feasibility of implementing the intervention in standard practice will guide recommendations for addressing reporting requirements while also assuring the primary goal of engaging clients in treatment at the first contact.

### Study aims

The primary aim of this trial is to determine the clinical effectiveness of MII intervention compared to intake-as-usual (IAU) on outcome variables of client entry, treatment attendance, and completion of an outpatient addiction treatment program. Two mechanisms of client engagement will be examined—changes in client motivation and client perception of the therapeutic alliance. The secondary aim of this trial is to determine the feasibility of implementing a motivational intervention across outpatient addiction treatment programs, based on quantitative and qualitative feedback from addiction treatment personnel. The Consolidated Framework for Implementation Research (CFIR) [32] will guide the approach of the implementation aim with a goal of identifying determinants of effective, sustained implementation of MII across the five broad CFIR domains: intervention characteristics, outer setting, inner setting, characteristics of individuals involved, and implementation process. Personnel will complete quantitative measures of organizational readiness and capability to change the intake process before and after the clinical trial ends, as well as a CFIR-guided individual interview after the clinical trial ends to identify specific facilitators and barriers to implementing the MII intervention into standard practice.

## Methods

### Study design

Funded by the National Institute on Drug Abuse (NIDA) as part of an initiative to improve behavioral healthcare services, the MII trial uses an HEI-1 design, where the primary outcome is clinical effectiveness, and the secondary outcome is implementation feasibility [33]. The study will be sufficiently powered to detect our primary outcome, which is the impact of the intervention on client entry in the outpatient treatment program, as well as the number of treatment sessions attended and whether client participants completed the three-month program (our secondary outcomes).

**Fig. 1:**
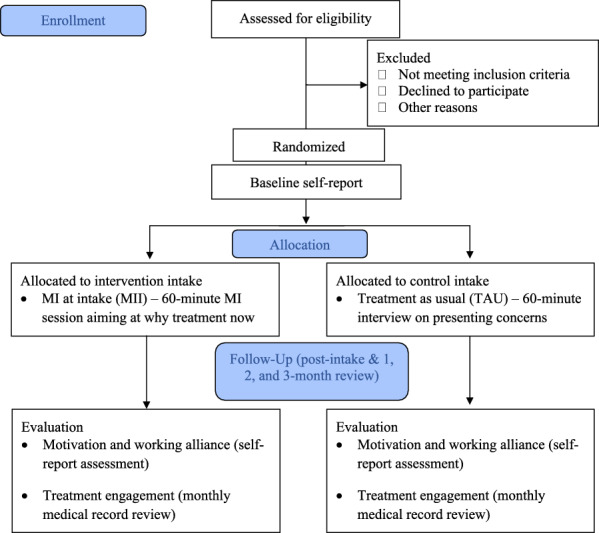
Clinical effectiveness trial diagram

**Fig. 2:**
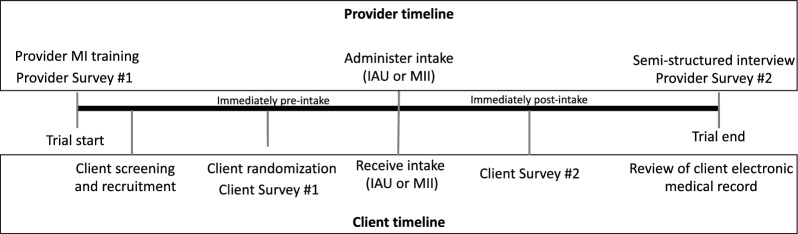
Client and personnel participant study timelines

To determine clinical effectiveness, a single-blind randomized clinical trial (RCT) will be used at two community-based addiction treatment programs—one that provides in-person services and one that provides telehealth services. Eligible clients will be those scheduled for an initial intake to the outpatient programs at each site. Interested participants will be randomly assigned to receive MII or intake-as-usual (IAU) by the on-site, clinical intake providers (see Fig. 1, CONSORT diagram). To determine implementation feasibility, a mixed-methods pre-post design will be used to evaluate changes in readiness and capability to change the intake process among addiction treatment personnel at both clinical sites. Specifically, we will administer a survey comprising several measures of implementation determinants to personnel before and immediately following the RCT, as well as an individual interview after the RCT to identify key facilitators and barriers to implementation effectiveness. To understand the feasibility of changing the intake process across addiction treatment programs, we also will administer the survey and individual interview to addiction treatment personnel employed at other substance treatment programs in New Mexico (see Fig. 2 for client and personnel study procedures).

The treatment protocol was approved by the University of New Mexico Institutional Review Board and is registered on ClinicalTrials.gov (NCT05489068). Electronic consent is required for personnel participation, and informed consent is required for client participation in the study.

### Study setting

The MII trial is being conducted in two community-based addiction treatment programs, one based in New Mexico and the other based in California. These sites provide outpatient treatment services to adults with SUD. An email detailing the MII trial and requesting participation as a study site was sent to 25 eligible addiction treatment facilities. Five facilities agreed to allow staff to be contacted by researchers for the implementation portion of the trial. However, three of the facilities were unable to recruit clients for the randomized clinical trial due to competing demands (e.g., implementing changes to other treatment services). The two participating treatment facilities are community-based addiction treatment programs that have similar intake procedures for clients seeking outpatient services, though one site provides in-person services and the other provides telehealth services.

### Study participants: recruitment, eligibility, randomization, and blinding

Client participants will be recruited from among individuals scheduled for a standard intake for the outpatient program (OP) at either of the two study sites. As part of standard procedures at both study sites, clients contact the treatment facility and complete a brief phone screener to identify their presenting concerns (i.e., substance use and mental health issues) and determine their level of care (e.g., detoxification services, OP), and are then scheduled for the intake appointment. Across both facilities, the intake appointment for clients seeking OP is the same. Clients who are scheduled for an OP intake will be recruited for study participation upon arrival to their scheduled intake appointment. Clients will be deemed eligible if they are at least 18 years old and English-speaking. Clients will be excluded from participation if they are experiencing severe withdrawal symptoms or are experiencing current symptoms of psychosis, mania, or suicidal intent. Exclusion criteria are determined through standard enrollment procedures at each site, such that clients first meet with administrative staff to complete consent for treatment paperwork and answer questions about their current need for detoxification services.

A research assistant will meet with eligible and interested participants to review the consent form, answer any questions, and assure the client that participation is not a requisite for concomitant care. Consenting client participants will be randomized to MII or IAU and randomization will be stratified by gender. The randomization sequences will be implemented (and concealed) in Redcap, a secure, customizable clinical trials management system used for data management and performed by a member of the research team who will not be involved in data collection and analytic procedures. Therefore, the principal investigator, research staff who will be conducting the informed consent and assessments, and all co-investigators will be masked to the intervention assignment. Only the designated project manager, one research assistant not involved in assessments or analyses, and the study intake therapists will not be masked to condition.

Addiction treatment personnel also will be included as participants to evaluate implementation feasibility. Personnel participants will comprise two groups: study-involved and study-naïve personnel. Study-involved personnel will be eligible to participate if they are English-speaking adults currently employed at one of the two study sites. Study-naive personnel will be eligible to participate if they are English-speaking adults who are currently employed at an addiction treatment program in the state of New Mexico. Study-involved personnel will be recruited from the two study sites but excluded from participation if they currently hold a leadership position (i.e., owner or clinical director). Study-naïve personnel will be recruited via email from treatment agency directors across the state of New Mexico and excluded if their job responsibilities include providing medical or administrative services. No manipulation or randomization procedures are in place for personnel participants. Whereas study-involved personnel participants will be asked to complete all implementation procedures, including pre-MII training assessments, optional MII training, and post-trial assessments and interview, the study-naïve personnel will be asked to complete only post-trial implementation procedures (i.e., post-trial assessments and interview).

### Sample size and power analysis

An intent to treat approach will be employed such that participant data will be analyzed based on the randomization assignment status, regardless of levels of engagement in the treatment program. Target enrollment of client participants is 150, with approximately 75 in the MII condition and 75 in the IAU condition. Power analyses based on a meta-analysis of 42 studies that evaluated client engagement following an MI intervention suggest that 75 participants per condition will provide sufficient power (0.80) to detect a medium effect size (*d* = 0.24 [CI 0.17, 0.31]) [34]. Given that client participant consent, baseline, follow-up assessments, and assigned intake condition will occur within a single visit, and that primary outcomes are based on electronic medical record review, we did not account for potential attrition.

### Interventions

In this trial, we are testing a stand-alone (“pure”) Motivational Interviewing at intake (MII) intervention. MI offers the optimal platform at intake because it uses a person-centered approach to explore client needs and elicit reasons the client would want to enter treatment, to understand prior successes or challenges managing substance use (e.g., past treatment attempts), and to reinforce the values and goals that motivated the client to seek treatment in the first place. This study seeks to *replace* an Information-First assessment as the first client contact with a clinician, instead offering an opportunity to improve substance use treatment engagement by using an evidence-based intervention in the manner that is theoretically most useful and as it is used with other health problems.

### Theoretical basis for MII

The proposed “pure” MII was developed in accordance with the theory of MI [35]. MI has been shown to increase client engagement and commitment to make behavioral changes [36–38]. Specifically, MI facilitates client behavior change by prioritizing client engagement. There are two active ingredients of pure MI that, when integrated and executed effectively, demonstrate how the therapeutic process promotes client decisions to make positive changes [31]. The relational component, based on Carl Roger’s client-centered therapy [39], involves a partnership between the therapist and client, wherein the therapist is demonstrating support, curiosity, and acceptance of the client’s current presentation. The technical component, based on behavioral reinforcement principles, entails how the therapist guides the conversation around change, eliciting client ambivalence around behavior change and reinforcing client statements that align with self-identified treatment goals. As such, the benefit of MI is based on how well the therapist leverages the therapeutic process to promote clients’ autonomous changes [40, 41]. Further, MI attends to the two notable therapeutic processes identified by both clients and providers as barriers to treatment engagement: client motivation and the therapeutic alliance. Client motivation is variable during the initial contact with a treatment agency, underscoring the need to elicit and reinforce client motivations for seeking treatment now. Also, the question-answer format of the standard intake assessment makes it difficult to develop therapeutic rapport, places therapists in the role of experts, and may disengage clients from treatment. The supportive yet directive nature of a pure MII is theorized to mobilize client motivation and therapeutic alliance and, thus, increase clients’ likelihood of entering treatment, staying in treatment, and completing treatment. This approach maximizes the empirical evidence and theoretical basis for designing an initial client contact where engagement is needed.

### MII condition

Client participants randomized to the MII condition will receive a 60-to-90-min pure MI session focused on why the client wants treatment at the current time. The MI session will follow the theory of MI and involve a goal-oriented and collaborative conversation about *why* the client wants treatment now, and how treatment might fit with client concerns, goals, and values. The provider will use open questions, reflective listening, and autonomy support in a flexible, non-authoritative manner. Rather than asking specific questions in different life domains, the provider will explore with the client their desires, abilities, reasons, and needs for treatment, how treatment fits with their values, and what successful treatment would look like to them. The client’s language about change will be elicited and reinforced strategically to increase its frequency and strength across the session. The goal of the MII conditions is to help clients resolve ambivalence around entering treatment now.

### IAU condition

Client participants randomized to the IAU condition will receive the 60-to-90-min standard clinic intake focused on obtaining a complete account of the client’s current and past substance use behavior and related sequelae. In line with ASAM guidelines, the assessment is a semi-structured interview that also assesses the client’s psychosocial history and clients are asked a series of questions related to support systems, living situation, education, occupational status, family, and medical history. The IAU is a comprehensive assessment that is delivered to all clients entering IOP at both study sites.

### Intervention training and monitoring

Some providers who conduct standard intake assessments at the participating study sites will be recruited to deliver the MII intervention. We will have separate intake providers deliver the MII and IAU conditions to interested participants. The MII intake providers will participate in a two-day MI training workshop (total of 8 h). The training includes a series of activities focused on increasing trainees’ knowledge and skills in the areas of client engagement (e.g., “A Taste of MI” exercise), therapeutic partnership (e.g., “Personal Values Card Sort” exercise), reflective listening (e.g., “Complexifying Reflections”), and client language (e.g., “Identifying Change Talk”). The workshop is comprised of educational materials and experiential exercises from the Motivational Interviewing Network of Trainers (MINT) Resources for Trainers. Following the workshop, MI intake providers will complete at least three individual feedback and coaching sessions, in accordance with recommended benchmarks of quality MI training [42, 43]. Each session will be scheduled after the intake providers complete a baseline audio-recorded clinical work sample with a standardized or actual patient. The work sample will be rated using the Motivational Interviewing Treatment Integrity (MITI) [44]. The clinical trial will begin after the MII intake providers reach proficiency on the MITI from these work samples. The MII intake providers also will participate in weekly supervision during the trial to assure ongoing adherence and competence to the MII intervention. The training workshop, feedback and coaching sessions, and weekly supervision will be provided by the first and second author, both of whom are licensed clinical psychologists and members of the MINT.

### Data collection protocol

The standard phone screen that is conducted with all clients requesting addiction treatment services at the two study sites will be used to collect client demographic characteristics, substance use and mental health information, as well as to determine client eligibility. The primary outcomes are treatment entry (i.e., attends first scheduled treatment session after the intake), number of treatment sessions attended, and completion (based on review of treatment attendance and discharge records) data obtained from electronic medical records, which will be collected each month following the intake and until clients are discharged from the treatment facility (standard treatment regimen: 3 months). A trained research assistant who is masked to allocated intake condition will administer brief self-report assessments before and after the intake condition to evaluate the theorized mechanisms of client engagement. Theorized mechanisms include change in client motivation, assessed with the Readiness Ruler (RR) [45] scores and perceived therapeutic alliance assessed using the Working Alliance Inventory (WAI) [46] scores. The RR is a measure of client readiness to change, with demonstrated psychometric support and good predictive validity [45, 47]. The WAI is a widely used measure of therapeutic alliance, with demonstrated psychometric support for a three-factor structure (goals, bond, and tasks), model invariance, and good internal consistency (αs = 0.85–0.90) [48]. Personal and medical information about participants will be protected by an NIH certificate of confidentiality.

### Implementation process evaluation

For the second objective—implementation feasibility—we will collect quantitative and qualitative data, in addition to evaluating intervention fidelity (described above), on the execution of the MII to inform implementation effectiveness and sustainability in community-based addiction treatment programs. Our mixed methods design is guided by the Consolidated Framework for Implementation Research (CFIR) [32]. A semi-structured interview will be the primary data source of implementation feasibility with study-involved and study-naïve personnel participants. Additionally, we will administer an online Qualtrics-based survey before and after the clinical trial ends to examine perspectives on the MII condition among clinicians and staff at both participating sites. For study-naïve personnel participants, they will be asked to complete the online survey one time, after the clinical trial ends. The online survey will comprise a brief demographic form to capture job characteristics (e.g., client caseload) and program characteristics (e.g., program location) and three validated measures—Texas Christian University-Organizational Readiness for Change (TCU-ORC), Readiness for Organizational Change (ROC), and Change Process Capability Questionnaire (CPCQ)—selected based on a systematic review of measures that capture implementation determinants across the five CFIR domains [49] (see Table 1). The TCU-ORC [50, 51] is the most widely used and well-validated measure of organizational readiness, and is comprised of 18 subscales across four broad areas: (1) motivation for change, (2) institutional resources of the program, (3) personality attributes of the staff, and (4) organizational climate of the program. The ROC [52] is a measure of organizational readiness and is comprised of four subscales: appropriateness, management support, change efficacy, and personally beneficial. Finally, the CPCQ [53, 54] is a measure of an organization’s capability to make changes, and is comprised of four subscales: previous history of change, plans for continuous organizational refinement, ability to initiate and sustain change, and change strategies.

**Table 1:** CFIR domains, definitions, examples, and data sources for implementation aim

Domain	Definition	Subconstruct examples	Data source
Intervention characteristics	Intervention attributes that may impact the success of implementation	Intervention source, adaptability, trialability, cost	ROC subscales map onto one subconstruct: relative advantage TCU-ORC subscales map onto one subconstruct: design quality and packaging CPCQ subscales map onto one subconstruct: relative advantage
Outer setting	Factors external to the organization that may impact intervention implementation	Patient needs and resources, external policies and incentives, peer pressure	TCU-ORC subscales map onto the following subconstructs: patient needs and resources, external policy and incentives, cosmopolitanism ROC subscales map onto one subconstruct: patient needs and resources
Inner setting	Internal factors that may impact the intervention implementation	Culture, implementation climate, compatibility, learning climate, available resources	TCU-ORC subscales map onto all subconstructs in the Inner Setting ROC subscales map onto the following subconstructs: implementation climate and readiness for change CPCQ subscales maps onto all subconstructs
Characteristics of the individuals involved	Knowledge, attitudes, perspectives, and traits of the individuals at the organization(s) involved in implementation	Knowledge and beliefs about the intervention, self-efficacy, other personal attributes	TCU-ORC subscales map onto the following subconstructs: self-efficacy and other personality attributes ROC subscales map onto the following subconstructs: knowledge and beliefs about intervention and self-efficacy
The process of implementation	Factors involved in the execution of intervention implementation	Planning, engaging, champions, reflecting and evaluating	TCU-ORC subscales map onto one subconstruct: planning CPCQ subscales map onto the following subconstructs: engaging, executing, reflecting/evaluating Individual semi-structured interviews with study-involved and -naïve personnel participants Intervention fidelity (using the MITI)

CFIR: Consolidated Framework for Implementation Research; TCU-ORC: Texas Christian University-Organizational Readiness for Change; ROC: Readiness for Organizational Change; CPCQ: Change Process Capability Questionnaire; MITI: Motivational Interviewing Treatment Integrity

### Outcomes

Clinical effectiveness is prioritized in the current study, and implementation feasibility is a secondary outcome. As such, the primary effectiveness outcome is whether clients enter the outpatient treatment program (i.e., attend their first scheduled treatment session). Secondary effectiveness outcomes include the number of treatment sessions attended and whether clients completed the outpatient treatment program. All effectiveness outcomes will be assessed using electronic medical record data and putative mechanisms of engagement will be assessed using self-report measures administered before and after the assigned intake condition. Mechanisms of effectiveness include change in client motivation and therapeutic alliance.

### Statistical analyses

#### Effectiveness outcomes

The primary effectiveness endpoints for this study will be medical record-verified client entry into treatment (completion of a 1st treatment session after the intake), number of treatment sessions attended, and client completion of treatment (i.e., based on review of treatment attendance and discharge records from assessor blind to intake condition). Analyses will be conducted using intent-to-treat principles [55], with all randomized participants included in the denominator for calculating engagement outcomes. Two binary logistic regression analyses will be used to compare the two intake conditions on the dichotomous outcomes of treatment entry and treatment completion. A negative binomial regression analysis will be used for the count outcome of number of treatment sessions attended. The following covariates will be included in each analysis: participant’s age, gender, race/ethnicity, substance use type, and pre-intake level of motivation. Further, we will explore possible therapist effects on each effectiveness outcome by performing one-way analyses of variance, specifying therapist as a random factor. We will obtain the percent of variance explained by the therapist by dividing the variance for the therapist factor by the total variance.

To evaluate mechanisms of effectiveness, separate mediation analyses will be performed on change in client motivation and therapeutic alliance on the three engagement outcomes. We will conduct logistic regression analyses for the two dichotomous outcomes and a negative binomial regression analysis for the count outcome. To explore whether observed direct effects are mediated by client motivation and therapeutic alliance, a bootstrapping method will be used (Preacher et al. 2008), providing a powerful test of mediation [56].

#### Implementation outcomes

Descriptive statistics, chi-square tests, and between-group analyses of quantitative measures will be performed. We will use a combined deductive and inductive thematic analysis approach [57] to qualitative data. Four raters will analyze qualitative data from interviews using NVivo software and in accordance with the six steps outlined by Braun and Clarke [57], and at least 50% of interviews will be double-rated to calculate inter-rater reliability. We will use deductive analysis to identify facilitators and barriers of MII implementation across the five CFIR domains. An inductive approach will then be used to generate themes and sub-themes from the qualitative data. Several analyses combining quantitative and qualitative data will be performed to fully conceptualize personnel perspectives on the intake process, to identify the most salient factors to implementation, and to determine which factors are common and unique across sites. Implementation data also will provide direction for a future implementation trial.

### Data safety management

Data collection procedures will be monitored by a Data Safety and Monitoring Board (DSMB) consisting of six clinical and experimental researchers independent from the sponsor and with no competing interests. DSMB members will receive bi-annual progress reports to determine whether to continue recruitment, propose a protocol amendment, or stop recruitment for the trial. Any adverse events will be reported to the principal investigator who will report it to the DSMB and NIDA in an annual progress report. The DSMB members will provide a recommendation on whether and how to proceed with the study. Serious adverse events and unanticipated problems will be reported within 24 h of occurrence to the institution’s IRB and to the study’s NIDA Program Officer in addition to the submission of a complete report.

## Discussion

The purpose of this trial is to determine the effectiveness of an MII intervention on client engagement and retention in outpatient addiction treatment. The MII trial also seeks to understand the potential for future sustainable implementation of MII across outpatient addiction treatment programs. As the field of addiction treatment has shifted focus to a recovery-oriented model of care, a wealth of person-centered treatment services focused on the whole person have been developed. In fact, extant research has revealed that services that better attend to client goals and values and also focus on non-substance related outcomes (e.g., well-being) predict client initial success and are better maintained over time [8].

The state of New Mexico implemented a Treat First model on January 11, 2016, which permits certified behavioral health facilities to delay the comprehensive intake assessment until the fourth session, allotting time for providers to develop rapport with clients and to facilitate client motivation to enter and remain in treatment. The Treat First model was developed with agreement by Medicaid and state-funded non-Medicaid funds to cover the cost of services without agencies needing to alter their billing process or being found noncompliant for not conducting the intake assessment and treatment plan during the first visit. The only requirement is that facilities become certified as a Treat First Agency (https://treatfirst.org/talks/how-to-become-a-treat-first-agency/). To date, 32 behavioral health agencies have been certified to use the Treat First Model, most of which are child or adult mental health treatment agencies. An evaluation of the Treat First model in its first year found that approximately 66% of clients came to all scheduled appointments. Although promising, no empirical investigations, to our knowledge, have looked at the utility of an evidence-based intervention during the first contact or its impact on organizations. By working with two addiction treatment programs that *do not* currently adhere to the Treat First Model, the current trial offers a comparative investigation of the utility of the standard comprehensive assessment versus an evidence-based intervention during intake, and thus can guide the dissemination of the Treat First Model to other treatment programs. Further, by simultaneously evaluating the feasibility of implementing an evidence-based intervention at intake, programs can identify ways to modify their intake procedures to engage clients while also adhering to state and federal agency requirements.

There are some limitations of the proposed study. First, clients allocated to the MII condition may be contacted by the study site 24 h after their intake to complete required paperwork for treatment enrollment. Most addiction treatment programs are required to collect certain client information at the outset for external agencies (e.g., funding agencies, health insurance companies). The additional contact to collect the required information may impact client engagement in treatment, though we hope the delay in collecting the information will help mitigate any negative impacts of the information collected and maximize the potential impact of the MII on client engagement outcomes. Second, it may be difficult to train study site intake providers to MI proficiency as evidenced in prior studies, and given their myriad of job duties (e.g., client caseload, administrative tasks, staff meetings) [31]. To address possible challenges with intake providers having the time to be trained in MI and/or to deliver the MI condition, licensed psychologists or advanced clinical psychology graduate students trained in the MI intake intervention will be available to serve as MI intake providers while study site intake providers will serve as intake-as-usual providers. The research team will train all study site personnel in MI either before or after the clinical trial, depending on whether study site intake providers are available serve as MI intake therapists.

This is the first trial to evaluate the effect of a pure MI intake intervention on treatment engagement in clients seeking outpatient addiction treatment programs and thus to provide evidence on how to reduce attrition in addiction treatment. Achieving study aims can demonstrate how aligning the theory and application of MI can improve clients’ entry and engagement in addiction treatment, a point of delivery that currently has the highest rate of attrition. If promising results are achieved, a larger, multisite trial with treatment programs across the United States can lead to external agencies approving the delay of standard intake assessment until clients are engaged and committed to treatment success.

## Data Availability

Data sharing not applicable to this article as no datasets were generated or analyzed during the current study.

## References

[1] Substance Abuse and Mental Health Services Administration(SAMHSA). Key substance use and mental health indicators in the United States: results from the 2020 national survey on drug use and health. HHS publication No PEP19-5068, NSDUH Series H-54. 2021;170:1–62.

[2] Section 1 PE tables–Results from the 2019 National Survey on Drug Use and Health: Detailed tables, SAMHSA, CBHSQ.

[3] National Institute of Drug Abuse. Principles of drug addiction treatment: A research-based guide (Third Edition). National Institutes of Health. Washington, DC; 2018.

[4] Loveland D, Driscoll H (2014). Examining attrition rates at one specialty addiction treatment provider in the United States: a case study using a retrospective chart review. Subst Abuse: Treat Prev Policy.

[5] Ball SA, Carroll KM, Canning-Ball M, Rounsaville BJ (2006). Reasons for dropout from drug abuse treatment: symptoms, personality, and motivation. Addict Behav.

[6] Substance Abuse and Mental Health Services Administration. National Survey of Substance Abuse Treatment Services (N-SSATS). : 2017. Data on Substance Abuse Treatment Facilities. Rockville; 2018.

[7] Mee-lee D. What ’ s new and how to use the ASAM criteria: skill-building in implementing the new edition May 12, 2014 9 AM – 4 : 45 PM A. Pretest questions. 2014;(c):1–28.

[8] Rush B, Urbanoski K. Seven core principles of substance use treatment system design to aid in identifying strengths, gaps, and required enhancements. 10.15288/jsads.2019.s18.9 PMC6377009 30681944

[9] Laudet AB, Stanick V, Sands B (2009). What could the program have done differently? A qualitative examination of reasons for leaving outpatient treatment. J Subst Abuse Treat.

[10] Palmer RS, Murphy MK, Piselli A, Ball SA (2009). Substance user treatment dropout from client and clinician perspectives: a pilot study. Subst Use Misuse.

[11] Allen RS, Olson BD (2016). Predicting attrition in the treatment of substance use disorders. Int J Mental Health Addict.

[12] Brener L, Resnick I, Ellard J, Treloar C, Bryant J (2009). Exploring the role of consumer participation in drug treatment. Drug Alcohol Depend.

[13] Hawkins EJ, Lott AMK, Malte CA, Frank AN, Hamilton B, Sayre GG (2017). Patients’ perspectives on care management services for complex substance use disorders. J Addict Dis.

[14] Ford Ii JH, Green CA, Hoffman KA, Wisdom JP, Riley KJ, Bergmann L et al. Process improvement needs in substance abuse treatment: admissions walk-through results. 10.1016/j.jsat.2007.02.003 PMC2151921 17499961

[15] Liu P, Currie S, Adamyk-Simpson J, Liu P, Currie S, Simpson A. What are the most important dimensions of quality for addiction and mental health services from the perspective of its users? PXJ. 2018;5(1):106–114.

[16] Pullen E, Oser C (2014). Barriers to substance abuse treatment in rural and urban communities: counselor perspectives. Substance Use and Misuse.

[17] DuPont RL, Compton WM, McLellan AT (2015). Five-year recovery: a new standard for assessing effectiveness of substance use disorder treatment. J Subst Abuse Treat.

[18] Dennis M, Scott CK (2007). Managing addiction as a chronic condition. Addiction Science Clinical Perspectives..

[19] McLellan AT, Meyers K (2004). Contemporary addiction treatment: a review of systems problems for adults and adolescents. Biol Psychiatry.

[20] McLellan AT, Carise D, Kleber HD (2003). Can the national addiction treatment infrastructure support the public’s demand for quality care?. J Subst Abuse Treat.

[21] Brown JM, Miller WR (1993). Impact of motivational interviewing on participation and outcome in residential alcoholism treatment. Psychology of Addictive Behaviors.

[22] Carroll KM, Libby B, Sheehan MJ, Hyland MN (2001). Motivational interviewing to enhance treatment initiation in substance abusers: an effectiveness study. Am J Addictions.

[23] Connors GJ, Walitzer KS, Dermen KH (2002). Preparing clients for alcoholism treatment: effects on treatment participation and outcomes. J Consult Clin Psychol.

[24] Dench S, Bennett G (2000). The impact of brief motivational intervention at the start of an outpatient day programme for alcohol dependence. Behav Cogn Psychother.

[25] Martino S, Carroll KM, O’Malley SS, Rounsaville BJ (2000). Motivational interviewing with psychiatrically ill substance abusing patients. Am J Addictions.

[26] Miller WR, Yahne CE, Tonigan JS (2003). Motivational interviewing in drug abuse services: a randomized trial. J Consult Clin Psychol.

[27] Donovan DM, Rosengren DB, Downey L, Cox GB, Sloan KL (2001). Attrition prevention with individuals awaiting publicly funded drug treatment. Addiction.

[28] Carroll KM, Ball Sa, Nich C, Martino S, Frankforter TL, Farentinos C (2006). Motivational interviewing to improve treatment engagement and outcome in individuals seeking treatment for substance abuse: a multisite effectiveness study. Drug Alcohol Depend.

[29] Burke BL, Arkowitz H, Menchola M (2003). The efficacy of motivational interviewing: a meta-analysis of controlled clinical trials. J Consult Clin Psychol.

[30] Miller WR, Rollnick S (2014). The effectiveness and ineffectiveness of complex behavioral interventions: impact of treatment fidelity. Contemp Clin Trials.

[31] Miller WR, Moyers TB (2017). Motivational interviewing and the clinical science of carl rogers. J Consult Clin Psychol.

[32] Damschroder LJ, Aron DC, Keith RE, Kirsh SR, Alexander JA, Lowery JC (2009). Fostering implementation of health services research findings into practice: a consolidated framework for advancing implementation science. Implement Sci.

[33] Curran GM, Bauer M, Mittman B, Pyne JM, Stetler C (2012). Effectiveness-implementation hybrid designs: combining elements of clinical effectiveness and implementation research to enhance public health impact. Med Care.

[34] Lundahl BW, Kunz C, Brownell C, Tollefson D, Burke BL (2010). A meta-analysis of motivational interviewing: twenty-five years of empirical studies. Res Social Work Pract.

[35] Miller WR, Rose GS (2009). Toward a theory of motivational interviewing. Am Psychol.

[36] Frost H, Campbell P, Maxwell M, O’Carroll RE, Dombrowski SU, Williams B (2018). Effectiveness of motivational interviewing on adult behaviour change in health and social care settings: a systematic review of reviews. PLoS ONE.

[37] Arkowitz H, Miller WR, Arkowitz H, Westra HA, Miller WR, Rollnick S (2015). Learning, applying, and extending motivational interviewing. Motivational interviewing in the treatment of psychological problems.

[38] Miller WR, Rollnick S, Miller WR, Rollnick S (2013). Motivational interviewing. Helping people change.

[39] Rogers CR (1959). A theory of therapy, personality, and interpersonal relationships as developed in the client-centered framework. Psychology: the study of science.

[40] Magill M, Apodaca TR, Borsari B, Gaume J, Hoadley A, Gordon REF (2018). A meta-analysis of motivational interviewing process: Technical, relational, and conditional process models of change. J Consult Clin Psychol.

[41] Villarosa-Hurlocker MC, O’sickey AJ, Houck JM, Moyers TB (2019). Examining the influence of active ingredients of motivational interviewing on client change talk. J Substance Abuse Treatment.

[42] Fu SS, Roth C, Battaglia CT, Nelson DB, Farmer MM, Do T (2015). Training primary care clinicians in motivational interviewing: a comparison of two models. Patient Educ Couns.

[43] Madson MB, Villarosa-Hurlocker MC, Schumacher JA, Williams DC, Gauthier JM (2019). Motivational interviewing training of substance use treatment professionals: a systematic review. Substance Abuse.

[44] Moyers TB, Rowell LN, Manuel JK, Ernst D, Houck JM (2016). The motivational interviewing treatment integrity code (MITI 4): rationale, preliminary reliability and validity. J Subst Abuse Treat.

[45] Hesse M (2006). The readiness ruler as a measure of readiness to change poly-drug use in drug abusers. Harm Reduct J.

[46] Horvath AO, Greenberg LS (1989). Development and validation of the working alliance inventory. J Couns Psychol.

[47] Maisto SA, Krenek M, Chung T, Martin CS, Clark D, Cornelius J (2011). A comparison of the concurrent and predictive validity of three measures of readiness to change alcohol use in a clinical sample of adolescents. Psychol Assess.

[48] Hatcher RL, Gillaspy JA (2006). Development and validation of a revised short version of the working alliance inventory. Psychother Res.

[49] Miake-Lye IM, Delevan DM, Ganz DA, Mittman BS, Finley EP (2020). Unpacking organizational readiness for change: an updated systematic review and content analysis of assessments. BMC Health Serv Res.

[50] Gagnon MP, Attieh R, Ghandour EK, Légaré F, Ouimet M, Estabrooks CA (2014). A systematic review of instruments to assess organizational readiness for knowledge translation in health care. PLoS ONE..

[51] Lehman WEK, Greener JM, Simpson DD (2002). Assessing organizational readiness for change. J Subst Abuse Treat.

[52] Holt DT, Armenakis AA, Feild HS, Harris SG (2007). Readiness for organizational change. J Appl Behav Sci.

[53] Rubenstein LV, Danz MS, Crain AL, Glasgow RE, Whitebird RR, Solberg LI (2014). Assessing organizational readiness for depression care quality improvement: relative commitment and implementation capability. Implement Science.

[54] Solberg LI, Asche SE, Margolis KL, Whitebird RR (2008). Measuring an organization’s ability to manage change: the change process capability questionnaire and its use for improving depression care. Am J Med Qual.

[55] Fisher LD, Dixon DO, Herson J, Frankowski RK, Hearron MS, Peace KE, Peace KE (1990). Intention to treat in clinical trials. Statistical issues in drug research and development.

[56] Fritz MS, MacKinnon DP (2007). Required sample size to detect the mediated effect. Psychol Sci.

[57] Braun V, Clarke V, Cooper H (2012). Thematic analysis. APA handbook of research methods in psychology.

